# Supplementary material for: Benzophenone and Benzoylphloroglucinol Derivatives from Hypericum sampsonii with Anti-Inflammatory Mechanism of Otogirinin A
Source: Molecules. 2020 Sep 28;25(19):4463. doi: 10.3390/molecules25194463 (PMC7582647; doi:10.3390/molecules25194463)
Supplement: Supplementary file 1 [file molecules-25-04463-s001.pdf]

Supplementary data

Benzophenone and benzoylphloroglucinol derivatives from *Hypericum sampsonii* with anti-inflammatory mechanism of otogirinin A

Chun-Yi Huang [a], Tzu-Cheng Chang [b], Yu-Jing Wu [c], Yun Chen [a], Jih-Jung Chen [a,d,*]

[a] *Faculty of Pharmacy, School of Pharmaceutical Sciences, National Yang-Ming University, Taipei, Taiwan*

[b] *Department of Forestry and Natural Resources, National Ilan University, Yilan City, Taiwan*

[c] *Institute of Pharmacology, National Yang-Ming University, Taipei, Taiwan*

[d] *Department of Medical Research, China Medical University Hospital, China Medical University, Taichung, Taiwan*

* Corresponding author at: Faculty of Pharmacy, School of Pharmaceutical Sciences, National Yang-Ming University, No.155, Sec.2, Linong Street, Taipei 11221, Taiwan

E-mail addresss: chenjj@ym.edu.tw (J.-J. Chen).

Contents

Fig. S1. HR-ESI-MS spectrum of **1**........S3

Fig. S2. ^{1}H NMR spectrum ($CDCl_3$, 500 MHz) of **1**........S3

Fig. S3. ^{13}C NMR spectrum ($CDCl_3$, 125 MHz) of **1**........S4

Fig. S4. ^{1}H-^{1}H COSY spectrum of **1**........S4

Fig. S5. NOESY spectrum of **1**........S5

Fig. S6. HSQC spectrum of **1**........S5

Fig. S7. HMBC spectrum of **1**........S6

Fig. S8. HR-ESI-MS spectrum of **2**........S6

Fig. S9. ^{1}H NMR spectrum ($CDCl_3$, 600 MHz) of **2**........S7

Fig. S10. ^{13}C-NMR spectrum ($CDCl_3$, 125 MHz) of **2**........S7

Fig. S11. ^{1}H-^{1}H COSY spectrum of **2**........S8

Fig. S12. ROESY spectrum of **2**........S8

Fig. S13. HSQC spectrum of **2**........S9

Fig. S14. HMBC spectrum of **2**........S9

Fig. S15. HR-ESI-MS spectrum of **3**........S10

Fig. S16. ^{1}H NMR spectrum ($CDCl_3$, 600 MHz) of **3**........S10

Fig. S17. ^{13}C-NMR spectrum ($CDCl_3$, 125 MHz) of **3**........S11

Fig. S18. ^{1}H-^{1}H COSY spectrum of **3**........S11

Fig. S19. ROESY spectrum of **3**........S12

Fig. S20. HMQC spectrum of **3**........S12

Fig. S21. HMBC spectrum of **3**........S13

Elemental Composition Report **Page 1**

Single Mass Analysis
Tolerance = 50.0 PPM / DBE: min = -1000.0, max = 1000.0
Element prediction: Off
Number of isotope peaks used for i-FIT = 2

Monoisotopic Mass, Even Electron Ions
31 formula(e) evaluated with 1 results within limits (all results (up to 1000) for each mass)
Elements Used:
C: 0-400 H: 0-1000 O: 4-4
HSa1R-P1 KE267 18-Apr-2018 15:51:19
0418_HSa1R-P1 22 (1.059) Cm (22-1x10.000) 2: TOF MS ES- 1.69e+003

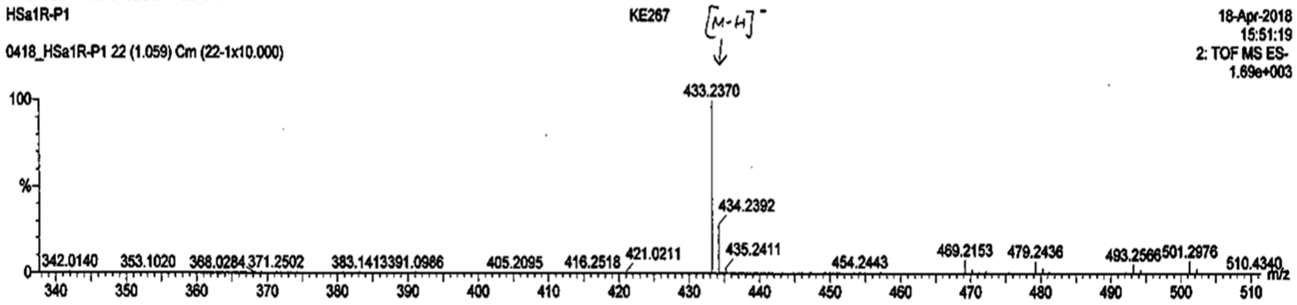

Minimum:				-1000.0			
Maximum:		5.0	50.0	1000.0			
Mass	Calc. Mass	mDa	PPM	DBE	i-FIT	i-FIT (Norm)	Formula
433.2370	433.2379	-0.9	-2.1	12.5	21.0	0.0	C28 H33 O4

Fig. S1. HR-ESI-MS spectrum of 1.

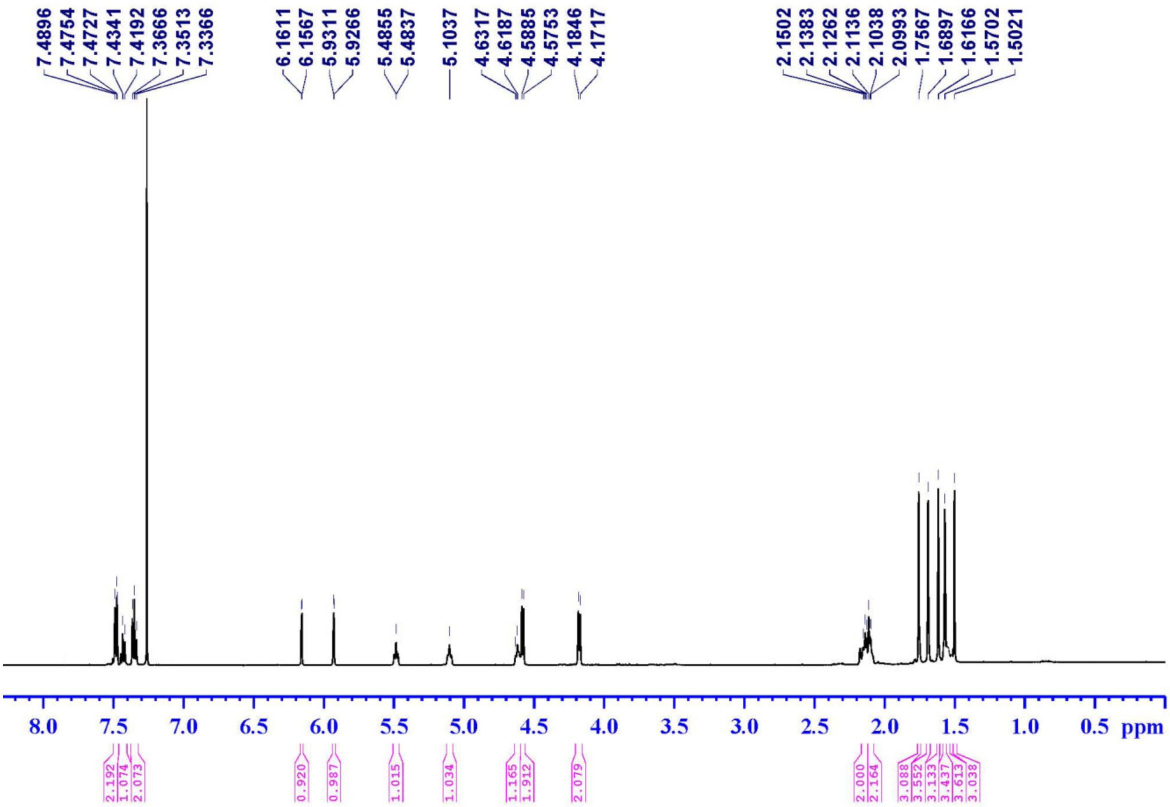

Fig. S2. ^{1}H-NMR spectrum ($CDCl_3$, 500 MHz) of 1.

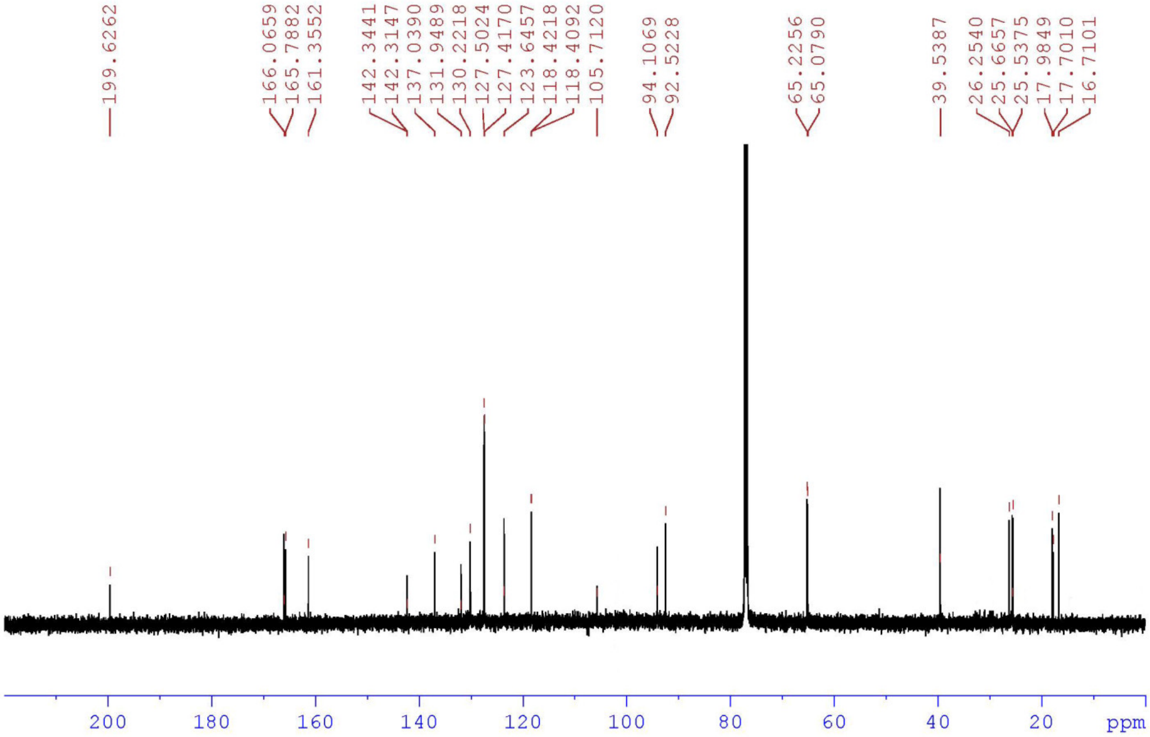

Fig. S3. ^{13}C-NMR ($CDCl_3$, 125 MHz) spectrum of 1.

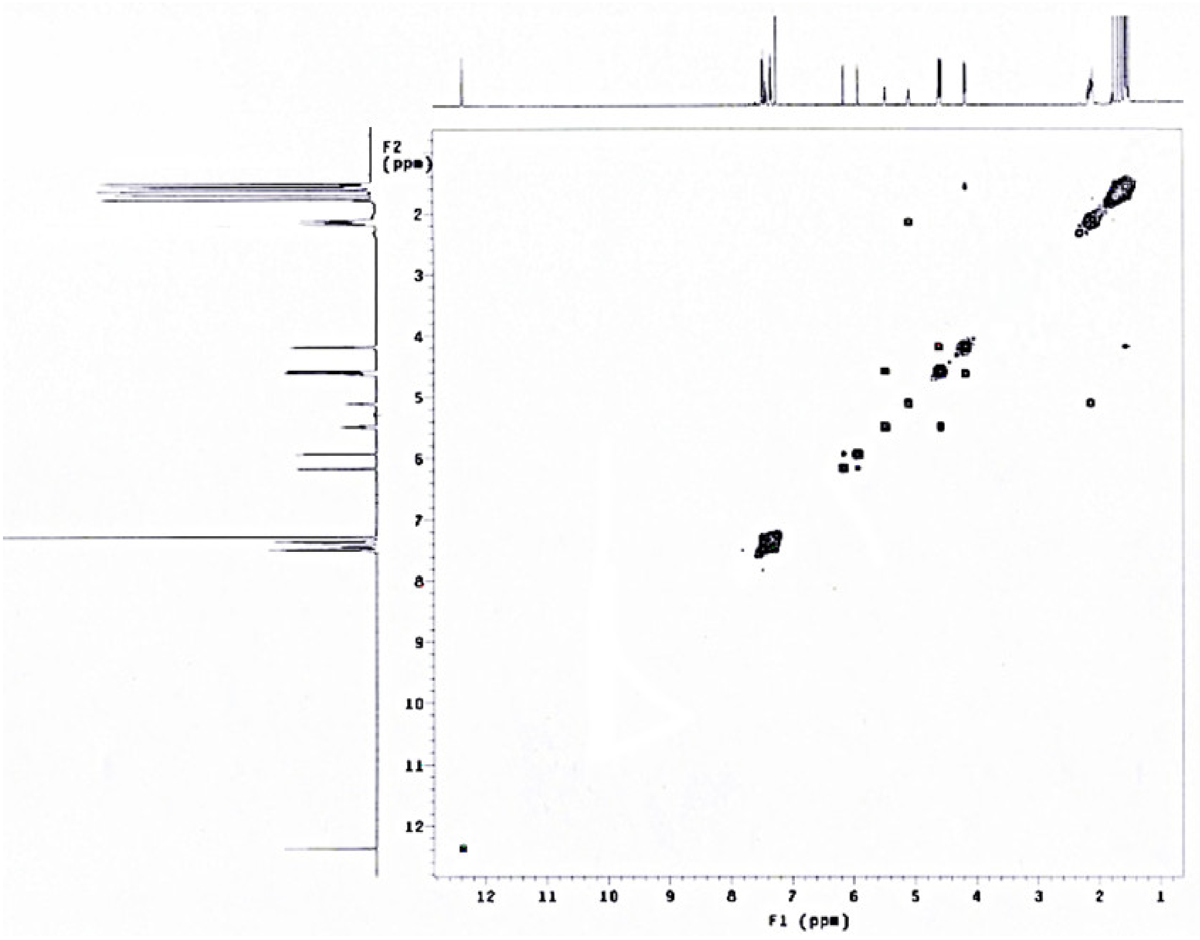

Fig. S4. ^{1}H–^{1}H COSY spectrum of 1.

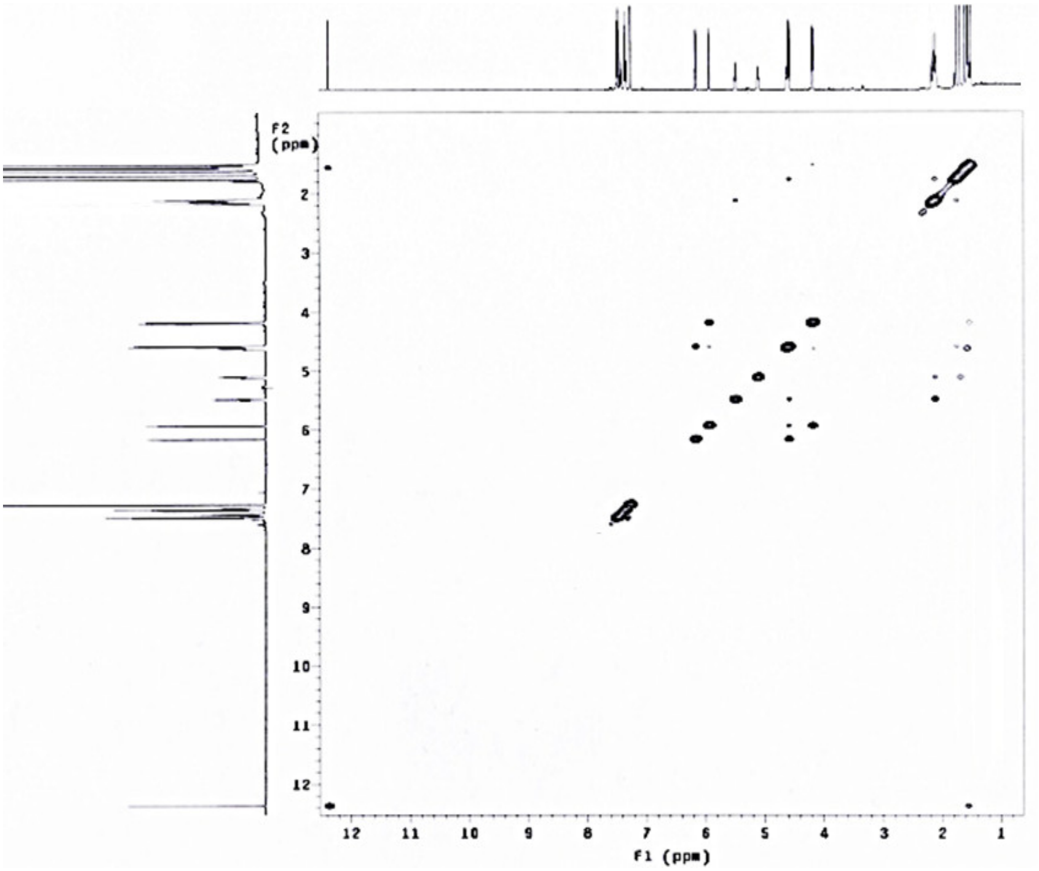

Fig. S5. NOESY spectrum of 1.

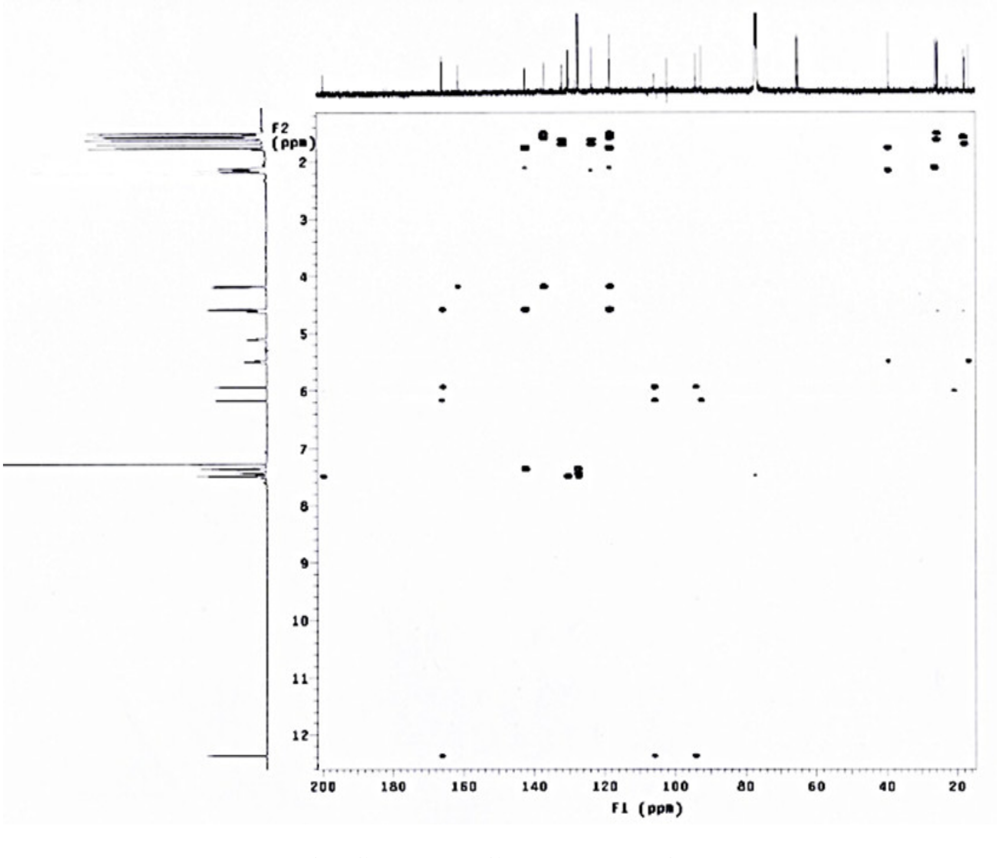

Fig. S6. HMBC spectrum of 1.

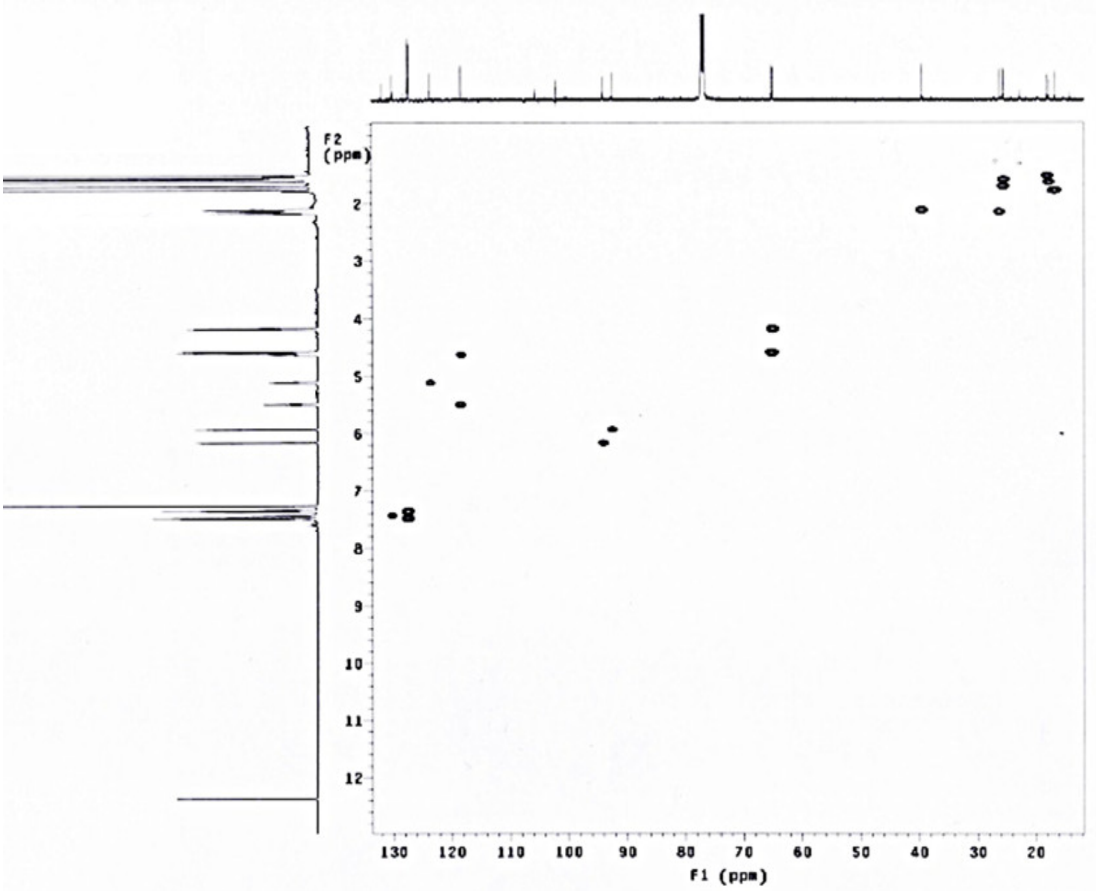

Fig. S7. HSQC spectrum of 1.

Charge number:1 Tolerance:100.00[ppm], 150.00 .. 150.... Unsaturation Number:-100.5 .. 200.0 (...
Element:^{12}C:32 .. 32, ^{1}H:0 .. 190, ^{23}Na:0 .. 1, ^{16}O:4 .. 4

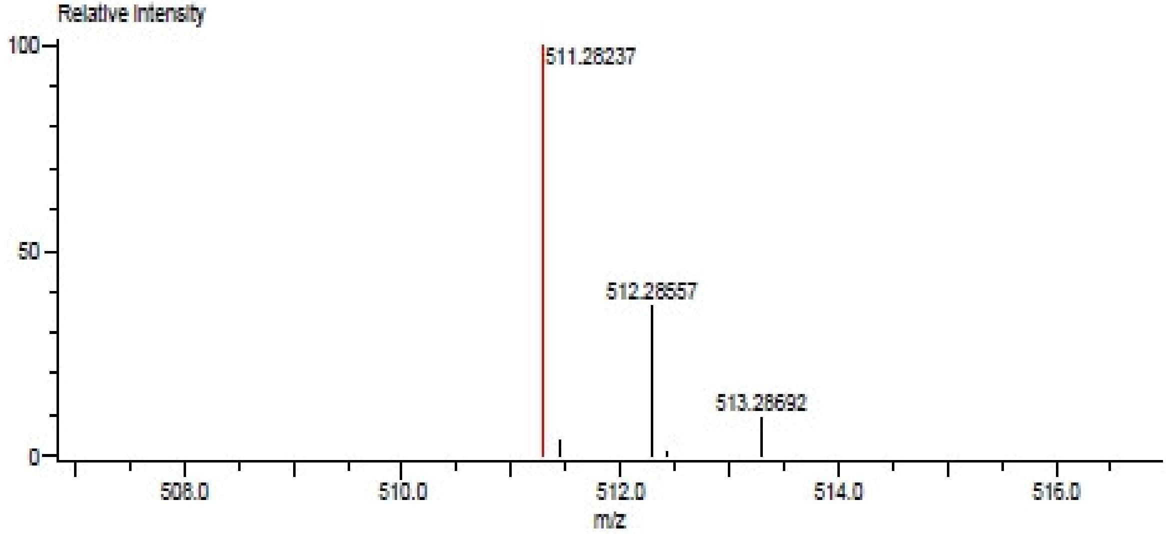

Mass	Intensity	Calc. Mass	Mass Difference [mDa]	Mass Difference [ppm]	Possible Formula
511.28237	24913.24	511.28243	-0.06	-0.12	$^{12}C_{32}{}^{1}H_{40}{}^{23}Na_{1}{}^{16}O_{4}$

Fig. S8. HR-ESI-MS spectrum of 2.

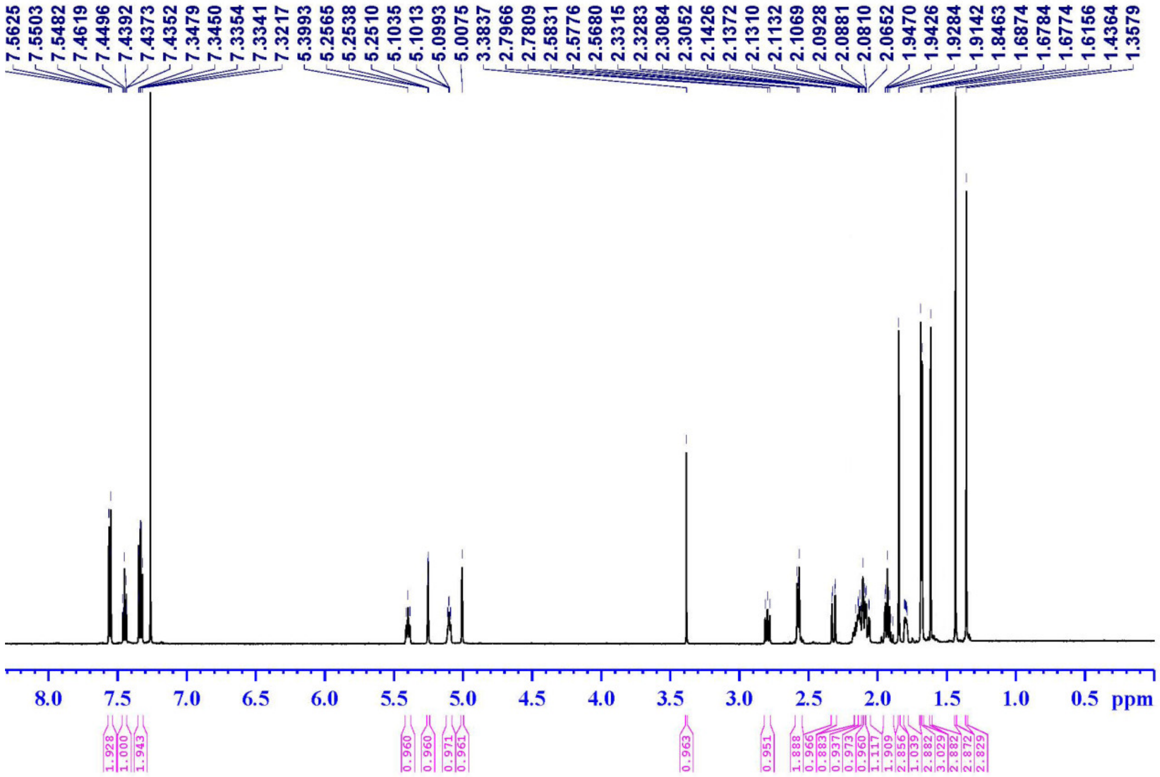

Fig. S9. ^{1}**H-NMR spectrum (CDCl**$_3$**, 600 MHz) of 2.**

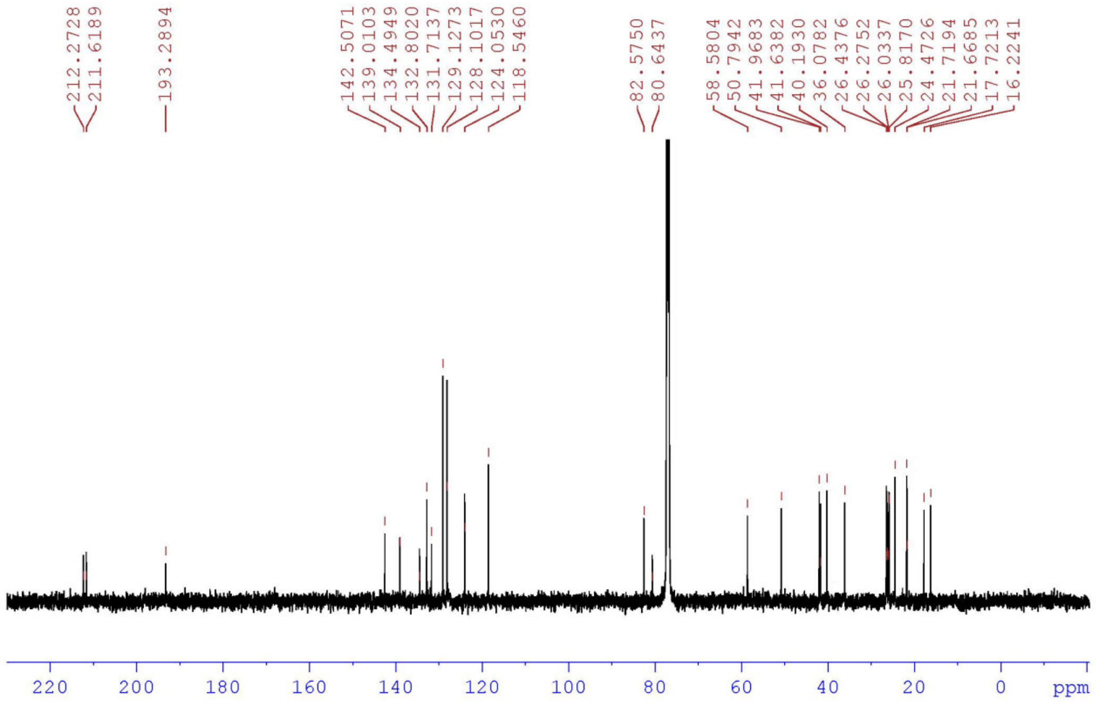

Fig. S10. ^{13}**C-NMR spectrum (CDCl**$_3$**, 125 MHz) of 2.**

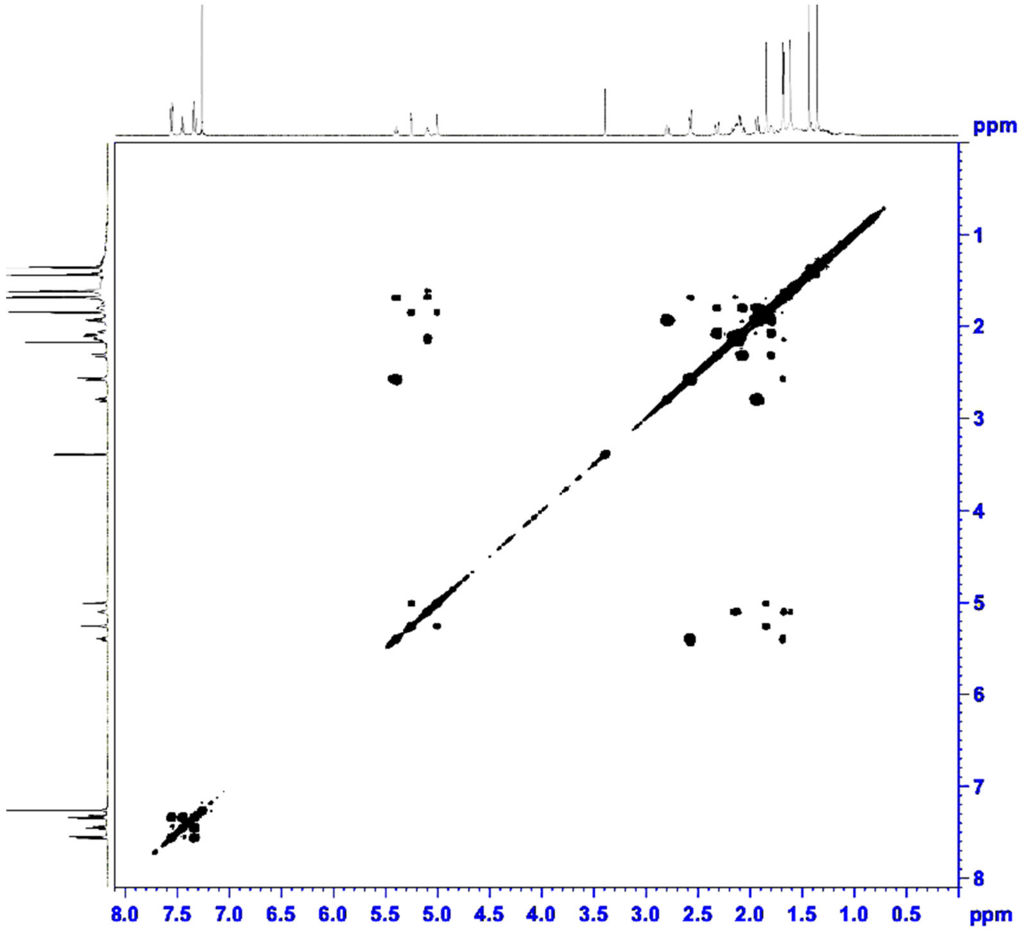

Fig. S11. ^{1}H–^{1}H COSY spectrum of 2.

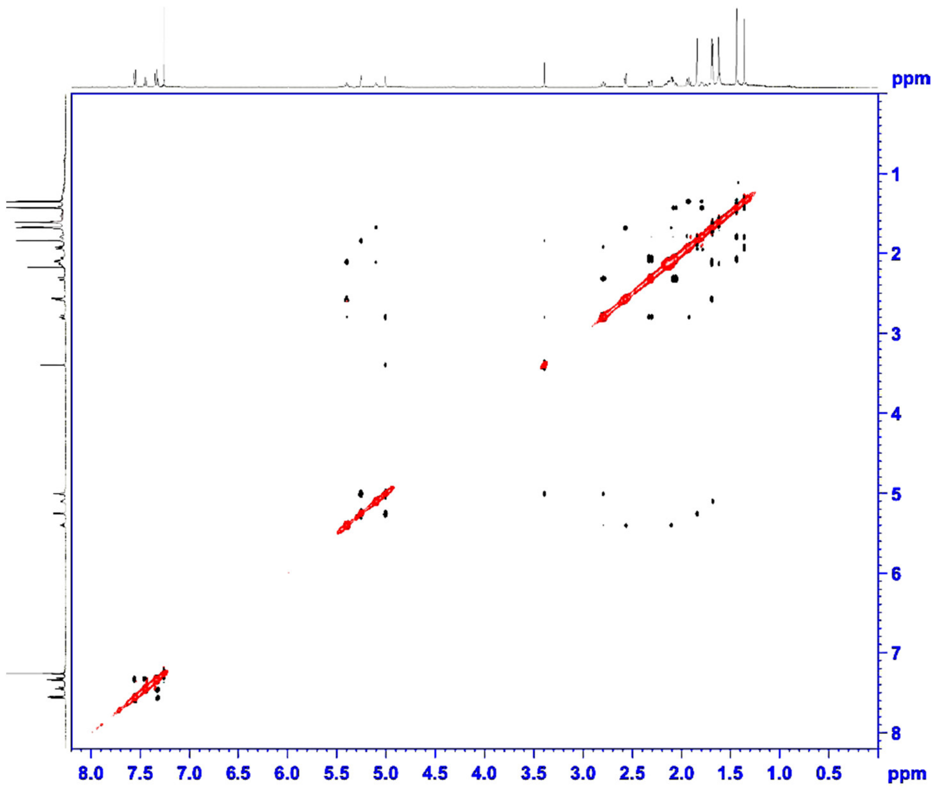

Fig. S12. ROESY spectrum of 2.

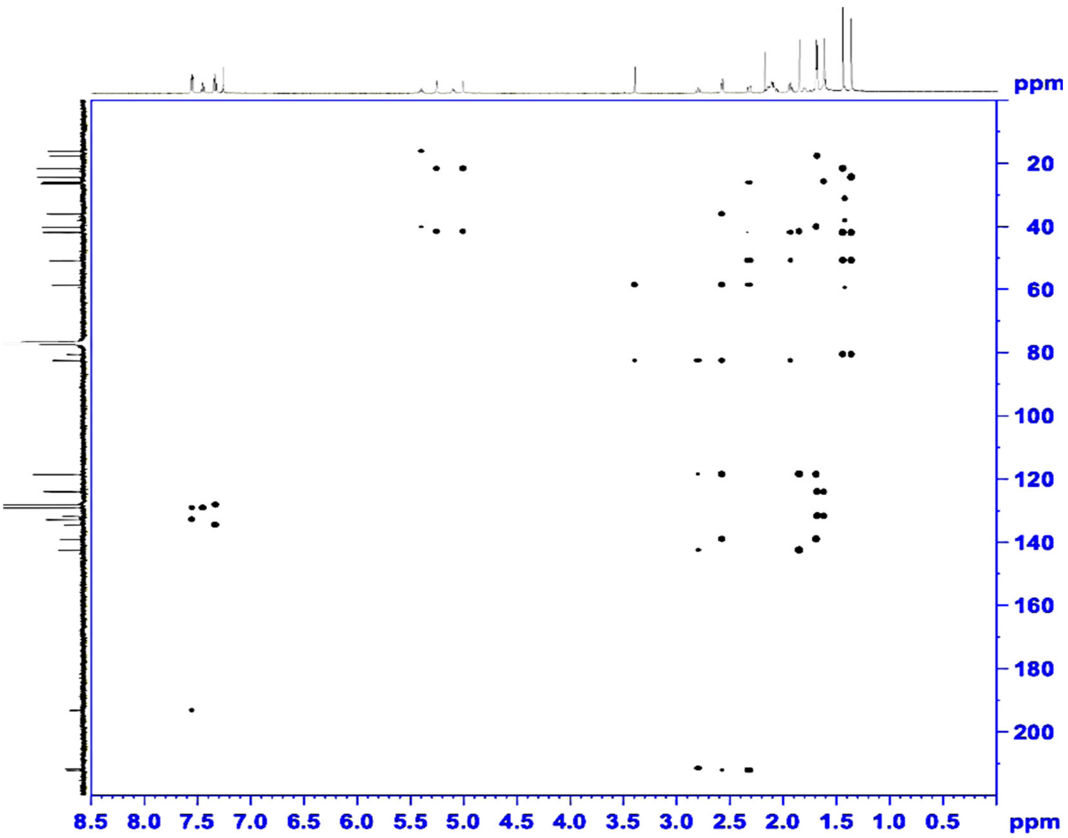

Fig. S13. HMBC spectrum of 2.

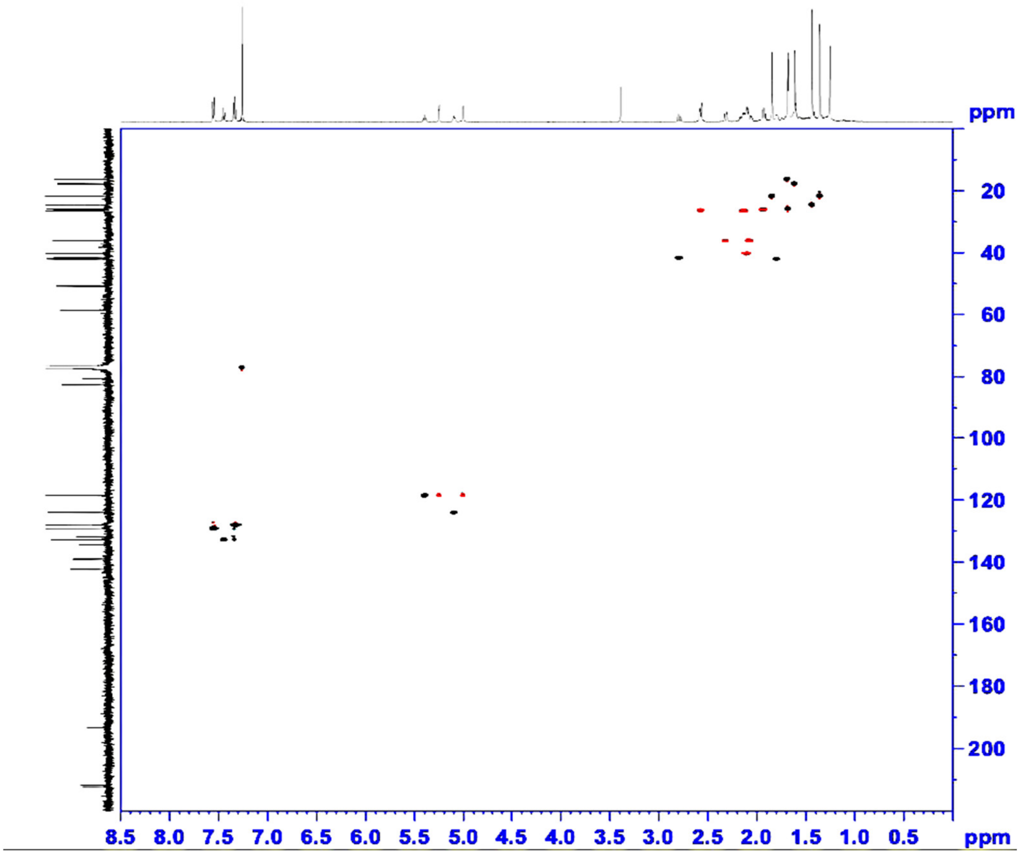

Fig. S14. HSQC spectrum of 2.

Data:MDP-3- 69
Comment:
Description:
Ionization Mode:ESI+
History:Average(MS[1] 0.60..0.66)

Acquired:6/3/2020 4:22:08 PM
Operator:AccuTOF
m/z Calibration File:20200102-1TFANa...
Created:6/3/2020 4:45:49 PM
Created by:AccuTOF

Charge number:1
Tolerance:250.00[ppm], 250.00 .. 250....
Unsaturation Number:-150.0 .. 200.0 (...
Element:^{12}C:30 .. 30, ^{1}H:0 .. 43, ^{23}Na:0 .. 1, ^{16}O:2 .. 2

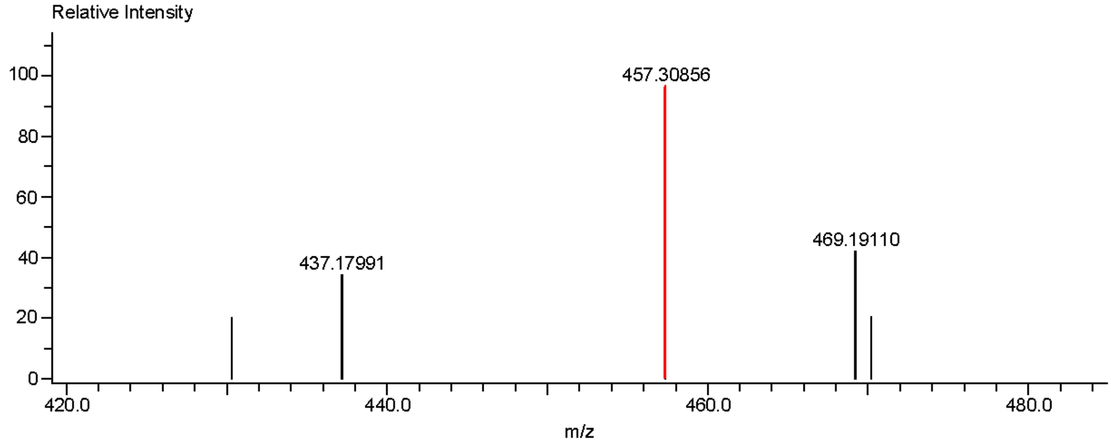

Mass	Intensity	Calc. Mass	Mass Difference [mDa]	Mass Difference [ppm]	Possible Formula
457.30856	21197.50	457.30825	0.31	0.62	$^{12}C_{30}{}^{1}H_{42}{}^{23}Na_{1}{}^{16}O_{2}$

Fig. S15. HR-ESI-MS spectrum of 3.

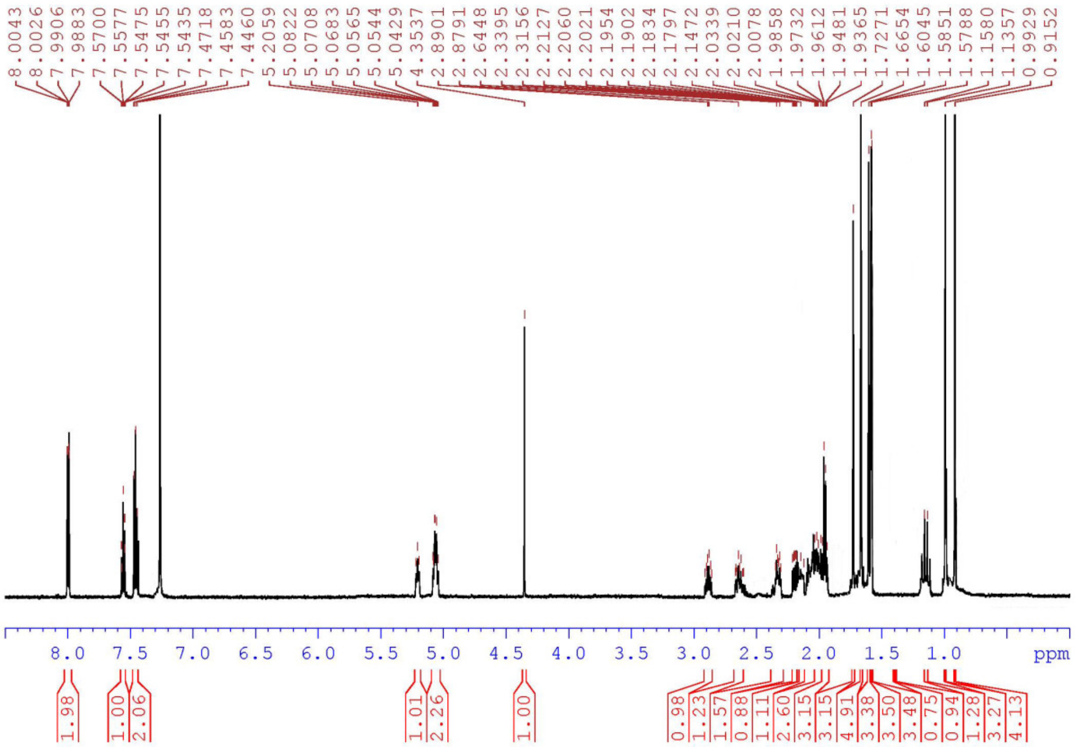

Fig. S16. ^{1}H-NMR spectrum ($CDCl_3$, 600 MHz) of 3.

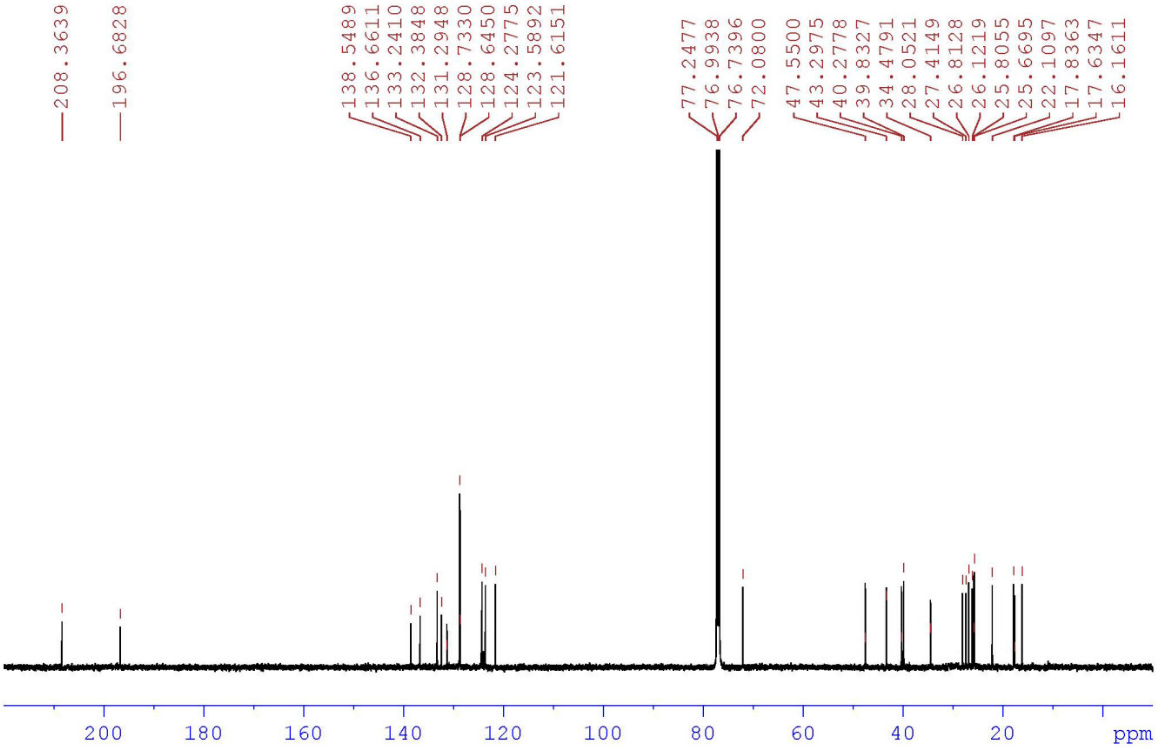

Fig. S17. ^{13}C-NMR spectrum of 3 ($CDCl_3$, 125 MHz) of 3.

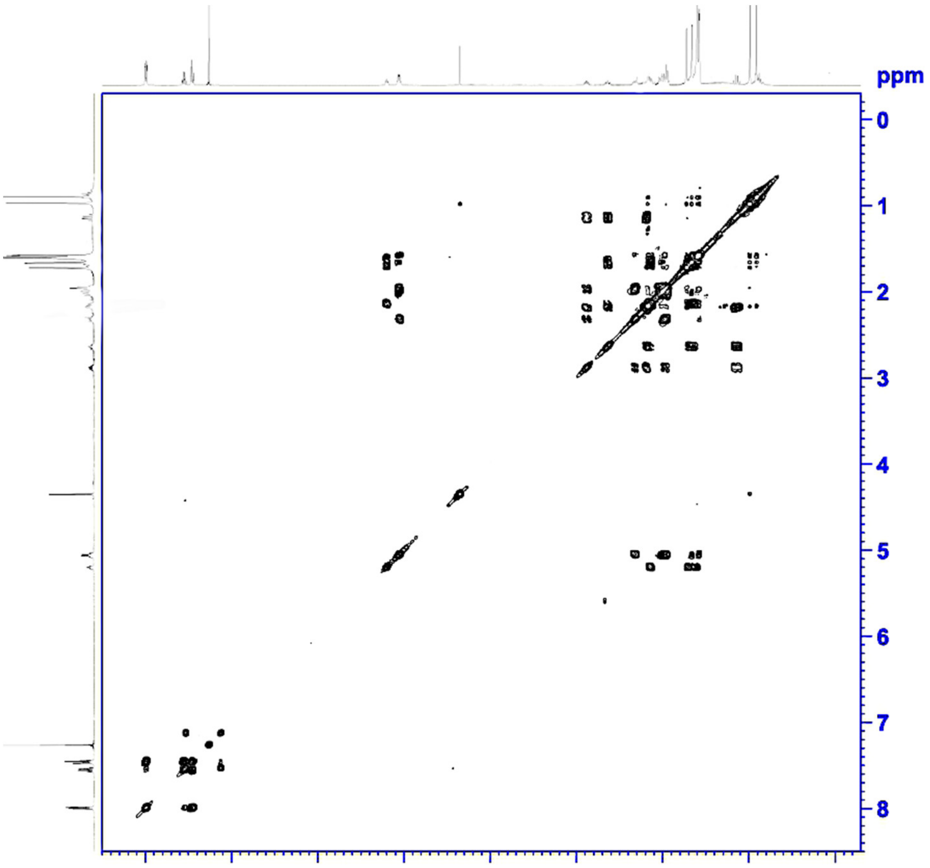

Fig. S18. ^{1}H–^{1}H COSY spectrum of 3.

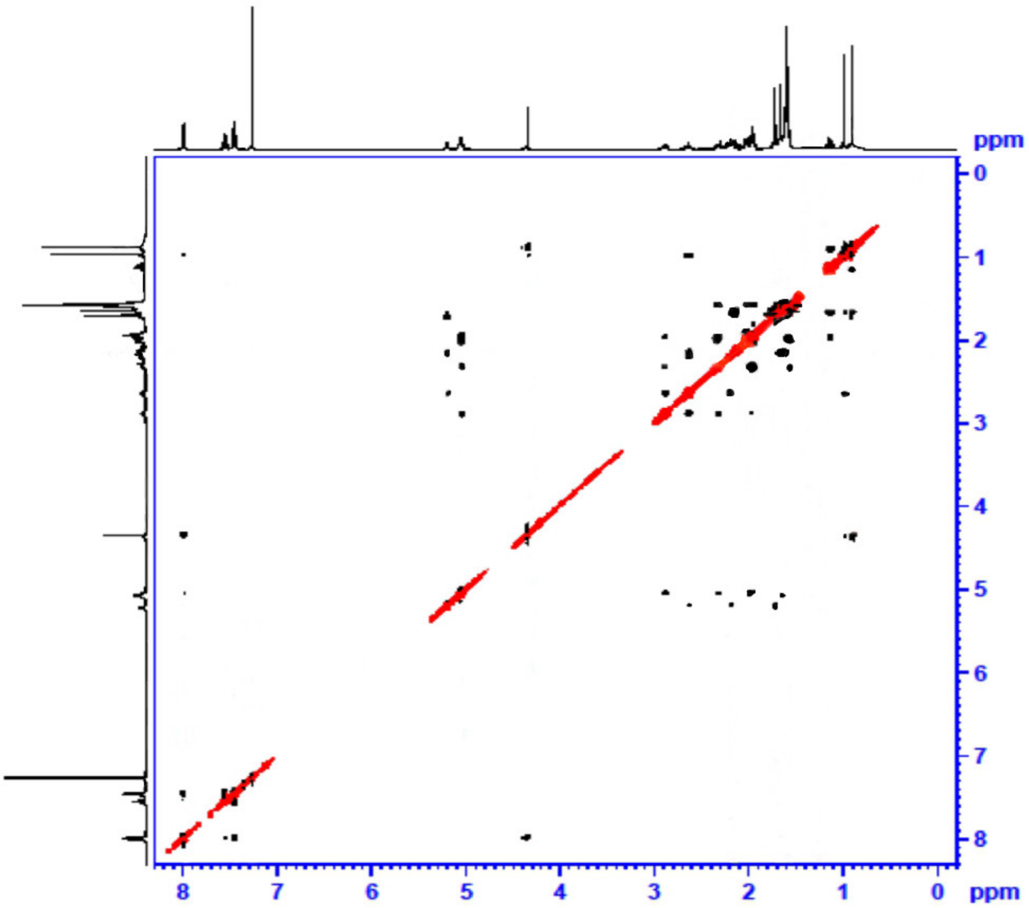

Fig. S19. ROESY spectrum of 3.

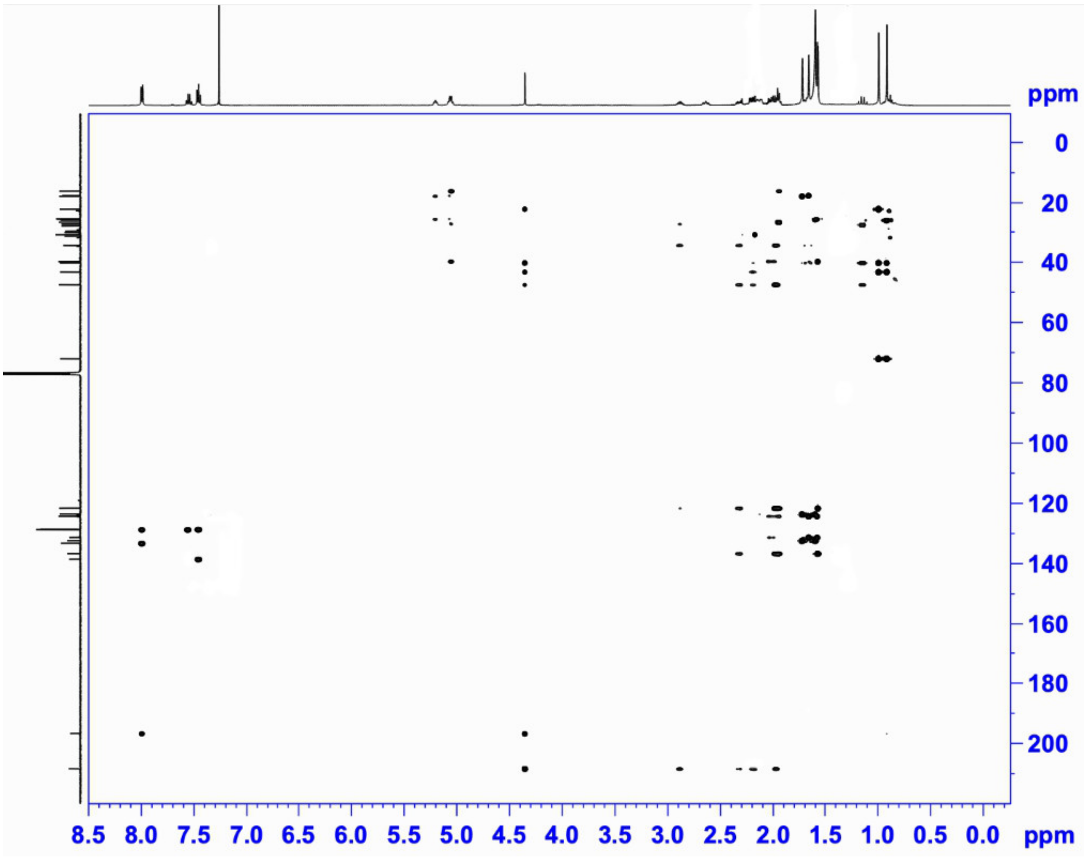

Fig. S20. HMBC spectrum of 3.

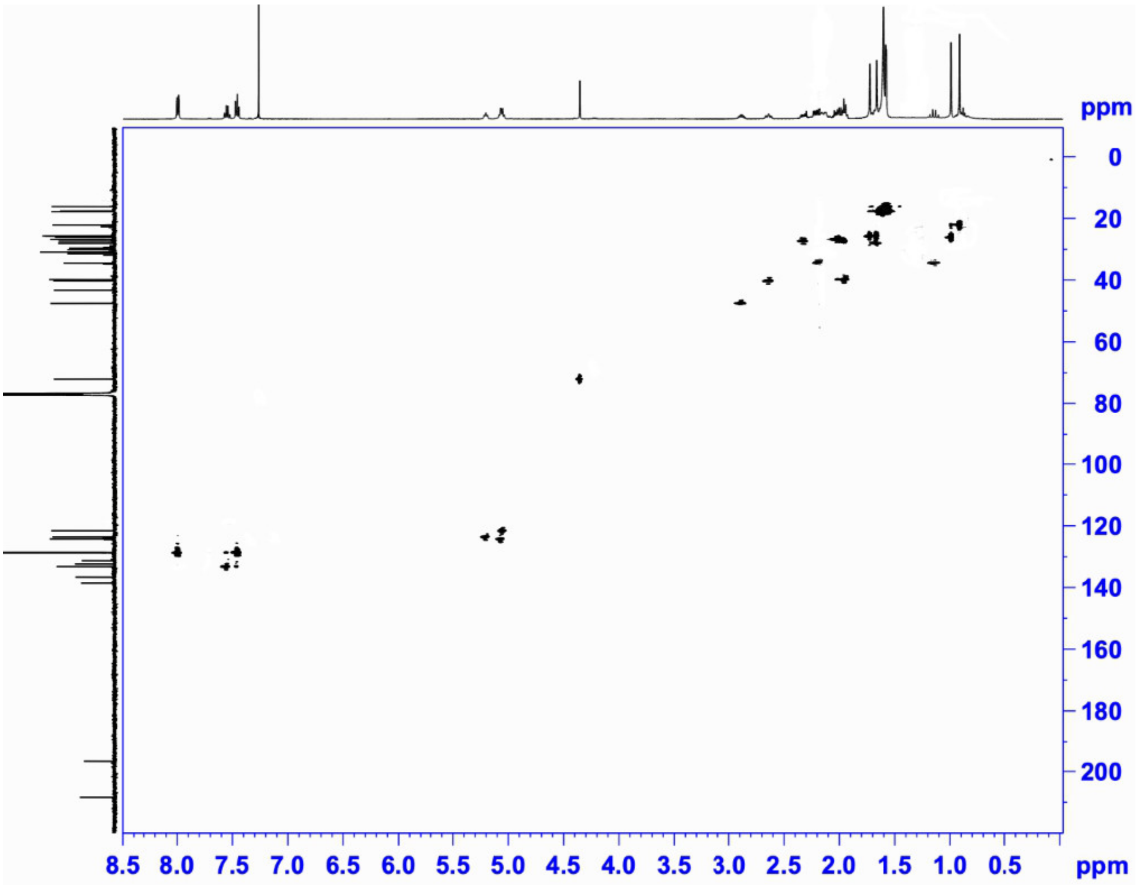

Fig. S21. HSQC spectrum of 3.
